# p62 limits Salmonella Typhimurium in macrophages through its role in cell signalling

**DOI:** 10.1099/acmi.0.001102.v3

**Published:** 2026-01-22

**Authors:** Daniel Underwood, Arda Balci, Virtu Solano-Collado, Heather M. Wilson, Massimiliano Baldassarre, Stefania Spanò

**Affiliations:** 1 School of Medicine, Medical Sciences and Nutrition, University of Aberdeen, Aberdeen, UK; 2 Institute of Medical Sciences, University of Aberdeen, Aberdeen, UK

**Keywords:** autophagy, macrophages, NFkB, p62, *Salmonella *Typhimurium

## Abstract

The intracellular autophagy receptor p62 (also known as Sequestosome-1) plays a dual role in autophagic flux and downstream Toll-like receptor signalling and has been implicated in modulating immune responses. However, its specific function in controlling intracellular bacterial survival, particularly in macrophages, remains less well characterized. *Salmonella enterica* serovar Typhimurium (*S*. Tm) is a major global pathogen and a leading cause of gastroenteritis-associated morbidity. We have previously demonstrated that host restriction of *S*. Tm in macrophages involves the GTPase Rab32 and the BLOC-3 complex. In the present study, we identify a novel interaction between p62 and Rab32. Notably, p62 restricts *Salmonella* survival independently of the Rab32/BLOC-3 pathway. Indeed, p62-knockdown in macrophages resulted in significantly increased intracellular bacterial survival, an effect that did not correlate with altered recruitment of canonical autophagy-related proteins, as assessed by fluorescence microscopy. Through real-time polymerase chain reaction (RT-qPCR) and infection assays, we further show that p62-depleted macrophages exhibit a dampened pro-inflammatory response, which corresponds with the increased bacterial burden. These findings provide new mechanistic insight into the role of p62 in modulating the macrophage inflammatory response during *Salmonella* infection, highlighting its contribution to host defence beyond its canonical functions in autophagy.

## Data Summary

All data are reported in this article and supplementary file.

## Introduction

The Gram-negative bacterium *Salmonella enterica* remains on the World Health Organization Bacterial Priority Pathogens List due to its high level of antibiotic resistance, endemic nature in low-income countries and significant global morbidity and mortality [1]. As a facultative intracellular pathogen, it can survive and replicate in a variety of host cells, including macrophages, by residing in a membrane-bound compartment known as the *Salmonella*-containing vacuole (SCV). Within the SCV, the bacterium utilizes a type-3 secretion system encoded in *Salmonella* pathogenicity island (SPI)-2 to translocate effector proteins that manipulate host cell processes, including intracellular trafficking and autophagy, thereby evading host antimicrobial responses [2].

The BLOC-3-Rab32 antimicrobial (BRAM) pathway restricts the growth of the human-restricted *S. enterica* serovar Typhimurium (*S*. Tm) in macrophages through trafficking of itaconate from the mitochondria to the SCV via Rab32 [3,4]. Nevertheless, the broad-host serovar *S*. Tm is able to overcome this pathway by releasing the effector proteins GtgE and SopD2 into the host cell, which target Rab32. Despite this, the SCV is at risk of xenophagy, a selective autophagy mechanism that specifically targets intracellular pathogens [5]. Indeed, Rab32 has also been shown to be necessary for autophagy to regulate the recycling of autophagosomal components [6].

Autophagosome formation around the target cargo requires specific adaptor proteins such as p62 (Sequestosome-1), which bridge ubiquitinated substrates to the autophagy machinery [7]. In non-professional phagocytic cells, autophagy has been shown to promote bacterial growth. However, in macrophages, p62 decorates the SCV and signals the xenophagy pathway [8,10]. p62 interacts with key components of the Toll-like receptor 4 (TLR-4) signalling pathway via its ubiquitin-associated, TRAF6-binding (TB) and zinc finger (ZZ) domains, which bind ubiquitin, TRAF-6 and receptor-interacting serine/threonine-protein kinase-1 (RIPK-1), respectively [11]. These interactions are likely dependent on K63-linked polyubiquitin chains, which are critical scaffolds for both TRAF6 and RIPK-1 signalling [12,13].

Although both Rab32 and p62 contribute to autophagy and the restriction of *Salmonella* in macrophages, it remains unclear whether they act within the same intracellular pathway. In this study, we provide evidence that the role of p62 in controlling intracellular *S*. Tm is independent of its function as an autophagy receptor. Instead, our data suggest that p62 is a critical downstream effector in the TLR-4 signalling cascade, promoting a pro-inflammatory response through activation of NFκB.

## Methods

### Cell and bacterial culture

Murine immortalized bone marrow-derived macrophages (iBMDMs) and HeLa cells were maintained in Dulbecco’s Modified Eagle Medium (DMEM) (Sigma-Aldrich) containing 10% FBS (complete medium) at 37 °C and 5% Carbon Dioxide.

The *S*. Tm strains SL1344 (WT) and SB2527 (**Δ***gtgE***Δ***sopD2*) were described previously [14,15]. mCherry-expressing variants were produced by transduction with P22 phages obtained from the *S*. Tm SL1344 *glmS::Cm::mCherry* (a gift from Knodler *et al.* [16]). These were cultured in/on Luria–Bertani (LB) broth/agar at 37 °C. Overnight cultures were diluted into 0.3 M Sodium Chloride-containing LB broth to induce expression of the SPI-1 type-3 secretion system and incubated until the culture reached an optical density (OD_600_) of 0.9.

### Plasmids

pBABE-FLAG-MCO-Rab32: The MCO (Mouse Codon Optimised)-Rab32 gene was amplified by PCR from the pUCIDT plasmid (IDT), digested with the restriction enzymes BamHI and EcoRI, and cloned into a pBABE vector.

pBABE-GFP-p62: The pMXs-GFP-p62 vector was used as a template to generate the retroviral vector expressing GFP-p62. Briefly, primers to amplify the GFP gene were designed to contain BamHI and SalI sites.

PCR was carried out using Phusion High-Fidelity DNA Polymerase (NEB). The PCR reaction mix was prepared as follows: 4 µl 5× HF Phusion buffer (NEB), 1.6 µl dNTPs (10 mM, Thermo Fisher), 1 µl 10 µM forward primer (Table 1), 1 µl 10 µM reverse primer, 10 ng of the plasmid DNA template and 0.2 µl Phusion polymerase topped up to 20 µl with nuclease-free water. The reaction was performed using an Eppendorf 5341 Mastercycler with a 30 s initial denaturation at 98 °C, followed by 30 cycles of extension and annealing consisting of 10 s at 98 °C, 10–30 s at 45–72 °C, 15–30 s per kilobase at 72 °C and a final extension of 5–10 min at 72 °C. Annealing temperatures were calculated using tmcalculator.neb.com and adjusted according to the primers used. After PCR amplification, the PCR product was digested and cloned into pBABE.

**Table 1.** List of primers used in this study

Primer	Sequence
f-GFP-p62	ATGCGGATCCGCCACCATGGTGAGCAAGGGCGAGG
r-GFP-p62	AGTCGTCGACTCACAATGGTGGAGGGTGC
f-MCO-Rab32	AGTCGGATCCATGGACTACAAAGACGACGACGACAAGGGCTCTATGGCCGGCGAAGGACTTGG
r-MCO-Rab32	ATGCTGAATTCAACAGCACTGGCCTTCTAGGC

The PCR products were purified using GENECLEAN II^®^ (MP) following the manufacturer’s instructions. Subsequent digestion was performed using appropriate restriction enzymes obtained from NEB; 3 µg of plasmid DNA or 1–2 µg of PCR product was digested with 1 µl restriction enzyme in 3 µl of 10× CutSmart buffer (NEB) in a final volume of 30 µl. Reactions were incubated at 37 °C in a water bath for 2 h. Following digestion, PCR products and plasmids were purified using GENECLEAN II^®^ (MP). Ligation reactions were carried out overnight at room temperature with T4 DNA ligase (NEB), with a reaction mix containing 2 µl 10× T4 ligase buffer (NEB), 1 µl T4 DNA ligase, 50 ng of plasmid or PCR product, in a final volume of 20 µl.

### Lentivirus production and transfection

Lentivirus particles were produced by co-transfecting 70% confluent HEK293-T cells with 9 µg of pLKO vector expressing the target short hairpin RNA (mSQSTM1_TRCN0000098619), along with 9 µg of pGag/Pol and pVSVG packaging plasmids, using polyethyleneimine (PEI) at a 3:1 PEI:DNA ratio. Twenty-four hours post-transfection, the medium was replaced with DMEM containing 30% FBS. Viral particles were collected 52 h post-transfection, spun down at 1,250 r.p.m. (Thermo O-G26) to remove cellular debris and frozen at −80 °C.

### Generation of p62 knockdown macrophages

MISSION^®^ shRNA (Sigma-Aldrich) were purchased as bacterial glycerol stocks and stored at −80 °C. Lentiviral particles expressing shRNAs were produced as described above, and iBMDM cells were infected with the virus at a 1:4 (virus:cells) ratio. After 24 h, the medium was replaced with medium containing puromycin (5 µg ml^−1^) for a minimum of 48 h to select transduced cells. Knockdown (KD) levels were determined by Western blot and quantitative PCR.

### Co-immunoprecipitation

HeLa cells were transfected with FLAG-Rab32 and GFP-p62 using PEI. Twenty-four hours post-transfection, cells were infected with *S*. Tm **Δ***gtgE***Δ***sopD2* for 3.5 h. Cells were then lysed in 0.5 ml of lysis buffer [(10 mM HEPES (Gibco), 150 mM NaCl (Merck), 1 mM EDTA (Sigma-Aldrich), 0.2% Triton X-100 (Sigma-Aldrich) and a protease inhibitor cocktail (Roche)]; anti-FLAG M2 agarose beads were added to the lysate, followed by incubation for 1 h on a rotary motor at 4 °C. Samples were then washed three times with lysis buffer, eluted using 2× SDS-loading buffer containing 10% β-mercaptoethanol, and Western blot analysis was carried out.

### SDS-PAGE Western blot

Cell samples were washed twice with PBS before being lysed with 2× Laemmli Blue buffer and then boiled at 95 °C. Polyacrylamide gels were prepared with the required percentage of polyacrylamide, depending on the weight of the protein being tested, followed by the addition of 10% ammonium persulphate and tetramethylethylenediamine to final concentrations of 0.15% and 0.001%, respectively.

Protein samples were transferred to a 12% polyacrylamide gel and run for 90 min (30 min at 80 V and 60 min at 120 V) with 3 µl of Colour Protein Broad Range protein ladder (NEB). Proteins were transferred to a PVDF membrane at 20 V for 60 min and blocked with 5% skimmed milk powder diluted in Tris-Buffered Saline + Tween (TBS-T) [137 mM NaCl (Merk), 20 mM Tris base (Sigma-Aldrich) and 0.05% (w/v) Tween-20 (Sigma-Aldrich)] for 1 h at room temperature.

All antibodies used are listed in Table 2 and were prepared in TBS-T. Incubation with primary antibodies was carried out overnight at 4 °C, and secondary antibodies were incubated at room temperature for 1 h.

**Table 2.** List of primary and secondary antibodies used for Western blots during this study

Antibody	Manufacturer	Raised in/against	Dilution
⍺-Rab32	Santa Cruz	Mouse/mouse	1:5,000
⍺-GFP	Invitrogen	Rabbit	1:5,000
FLAG	Invitrogen	Mouse	1:10,000
⍺–β-Actin	Cell Signalling	Rabbit/mouse	1:10,000
⍺-p62	Biotechne	Rabbit/mouse	1:2,000
⍺-p65 and pp65	Cell Signalling	Rabbit/mouse	1:1,000
⍺-Mouse/rabbit IgG – HRP	Sigma-Aldrich	Goat/mouse and rabbit	1:10,000

### Gentamicin protection assay

iBMDMs (1×10^5^ cells per well, 24-well plate) were infected with *S*. Tm at the indicated multiplicity of infection (MOI) and incubated for 1 h at 37 °C. Cells were then washed twice with Hank’s Balanced Salt Solution (HBSS), and complete medium containing 100 µg ml^−1^ of gentamicin was added. After 30 min, the medium was replaced with complete medium containing 5 µg ml^−1^ of gentamicin to prevent reinfection. These conditions were maintained until 30 min prior to each time point, where the medium was replaced with complete media containing 100 µg ml^−1^ gentamicin before lysis. Cells were lysed with a 0.1% Triton X-100 solution, and serial dilutions of lysates in PBS were plated onto LB agar plates and incubated at 37 °C overnight.

For experiments using conditioned media, confluent 10 cm plates containing WT and p62 KD iBMDMs were infected with *S*. Tm WT at an MOI of 10, using the method described above. At 24 h post-infection, supernatants were removed and filtered with a 0.22 µm filter to remove any bacteria and cellular debris. Samples were stored at 4 °C. On the day of the experiment, supernatants were mixed with fresh media containing 5 µg ml^−1^ gentamicin and used in a gentamicin protection assay.

### Immunofluorescence microscopy

Macrophages were seeded at a density of 1×10^5^ cells per well on glass coverslips and infected as previously described with *S*. Tm. Coverslips were washed twice with 1× PBS and permeabilized in 500 µl of permeabilization buffer (0.1% Triton X-100, 2% BSA, 50 mM Ammonium Chloride and 1× PBS) before being stained with the relevant antibody (Table 3).

**Table 3.** List of stains, primary and secondary antibodies used for fluorescent microscopy during this study

Antibody	Manufacturer	Raised in/against	Dilution
α-LC3	Proteintech	Rabbit/mouse	1:100
a-Rubicon	Proteintech	Rabbit/mouse	1:200
α-Rabbit 488 nm	Invitrogen A-11008	Goat/rabbit	1:2,000
α-Rab32	Santa Cruz	Mouse/mouse	1:2,000
Goat α-Mouse 647 nm	Invitrogen A-32728	Goat/mouse	1:2,000

### Real-time Polymerase Chain Reaction

RNA was extracted using the RNeasy kit (Qiagen), and total RNA concentration was measured using a NanoDrop [17,18]. cDNA was synthesized from total RNA using the iScript^TM^ cDNA synthesis kit (Bio-Rad) as per the manufacturer’s instructions, consisting of 1,000 ng of RNA mixed with 4 µl of 5× iScript™ reaction buffer and 1 µl of iScript™ reverse transcriptase in a final volume of 20 µl. The reactions were carried out as follows: 5 min at 25 °C to prime the reaction, 30 min at 42 °C for reverse transcription and 5 min at 85 °C to inactivate the enzyme.

The quantitative PCR reaction was carried out using a 96-well plate (Applied Biosystems), with a reaction mixture containing 2 µl of cDNA, 10 µl of SYBR Green mix (Takara), 0.1 µl of 100 mM forward and reverse primers (Table 4) and 7.8 µl of nuclease-free water. Thermal cycling was performed using StepOnePlus^TM^ (Applied Biosystems). The thermocycling protocol was as follows: 95 °C for 10 min, 40 cycles of 95 °C for 15 s and 60 °C for 1 min.

**Table 4.** Primers used for qPCR (5′−3′)

Gene	Forward	Reverse
*Il1a*	CGAAGACTACAGTTCTGCCATT	GACGTTTCAGAGGTTCTCAGAG
*Il1b*	GAAATGCCACCTTTTGACAGTG	TGGATGCTCTCATCAGGACAG
*Il1r1*	GTGCTACTGGGGTCATTTGT	GGAGTAAGAGGACACTTGCGAAT
*Il10*	CTTACTGACTGGCATGAGGATCA	GCAGCTCTAGGAGCATGTGG
*Tnfa*	GGTGCCTATGTCTCAGCCTCCT	GCCATAGAACTGATGAGAGGGAG
*Il6*	CTGCAAGAGACTTCCATCCAG	AGTGGTATAGACAGGTCTGTTGG
*Gapdh*	AGGTCGGTGTGAACGGATTTG	TGTAGACCATGTAGTTGAGGTCA

Each gene target was quantified in triplicate, and the housekeeping gene GAPDH was used as the control. Differential gene expression was quantified using the ΔΔCt method [19].

### Sandwich ELISA

TNFα and the IL-1β ELISA kits were purchased from Bio-Techne, and the manufacturer’s instructions were followed. Antibodies were coated onto a 96-well plate and left overnight at room temperature. Cell supernatants and a known standard curve were loaded onto the plate and left to incubate at 37 °C for 90 min. A biotin-conjugated antibody at the necessary dilution (TNFα at 1:10,000 and IL-1β at 1:20,000) was added and left to incubate at 37 °C for an additional 60 min. Diluted streptavidin-HRP (horseradish peroxidase) was added and incubated under the same conditions for 1 h. Premixed TMB (Thermo Fisher) was added to each well and left to incubate for 30 min at room temperature, or until a colour change was observed. Reactions were stopped with 0.2 M Sulphuric Acid, and absorbance was measured at 450 nm (Varioskan LUX; Thermo Fisher). Thorough washing with PBS+0.05% Tween-20 was performed between each step prior to the addition of TMB.

### Statistical analysis

Data from replicated experiments were analysed using GraphPad Prism 10. Student’s t-tests were used to compare differences between mean values of normally distributed data against a significance value of 0.05. A Mann–Whitney U test was used when the data were not normally distributed.

## Results

### p62 impacts *Salmonella* survival

**Fig. 1.**
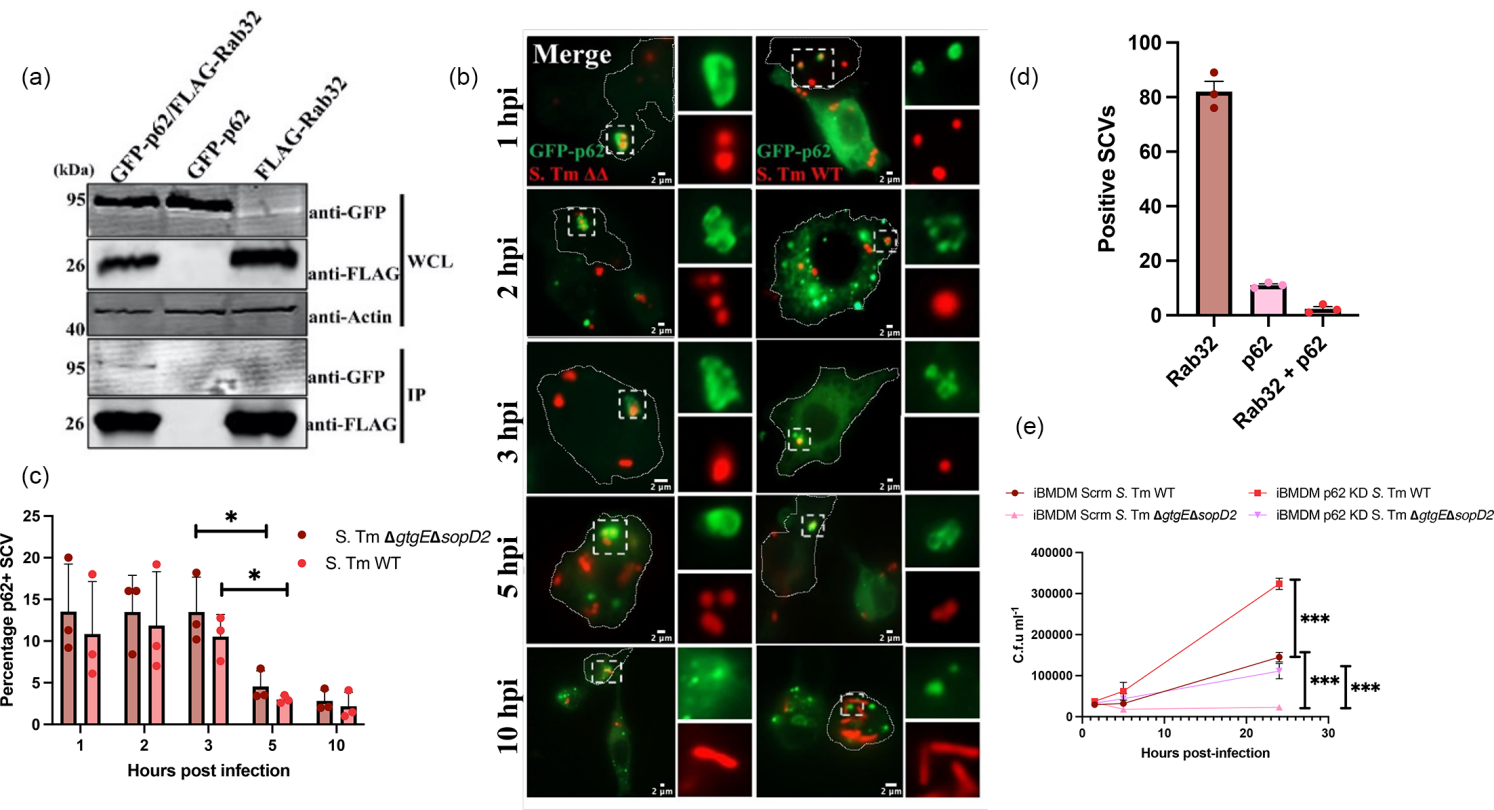
p62 and Rab32 are novel interacting partners but contribute to *Salmonella* killing by different pathways. (a) HeLa cells were transiently co-transfected with FLAG-Rab32 and GFP-p62 and lysed, and immunoprecipitation was carried out using anti-FLAG agarose beads. Samples were analysed by immunoblotting with anti-FLAG, GFP and actin antibodies (loading control) (*n*=2). (b) iBMDM cells were transduced with a retrovirus expressing GFP-p62. Cells were then selected with puromycin, infected with mCherry::*S.* Tm SB2527 (𝝙gtgE𝝙sopD2) or WT and fixed at the indicated time points. (c) Quantification of bacteria in p62-positive vacuoles at the times indicated is shown. Data are presented as mean±sd from three independent experiments, in which at least 100 bacteria were counted for each time point. (d) Quantification of bacteria in Rab32-, p62- or Rab32- and p62-positive vacuoles at 2.5 hpi is shown. Data are shown with a mean±sd of three independent experiments. (e) Control (Scrm) iBMDM and cells deficient in p62 were infected with *S.* Tm WT or SB2527 (𝝙gtgE𝝙sopD2) and lysed at the indicated time points. Lysates were plated on LB agar, and colonies were counted. Values are means±sem of three independent experiments. ***P<0.0005 (Student’s t-test). hpi, hours post-infection; IP, immunoprecipitate; WCL, whole cell lysate.

Proteomics analysis of FLAG-Rab32 immunoprecipitates from *Salmonella*-infected macrophages identified p62 as a potential interacting partner of Rab32 [20]. To validate this interaction, HeLa cells were co-transfected with FLAG-Rab32 and GFP-p62, followed by infection with *S*. Tm strain **Δ***gtgE***Δ***sopD2*. Co-immunoprecipitation using anti-FLAG M2 agarose beads revealed that GFP-p62 co-precipitated with FLAG-Rab32. Western blot analysis confirmed the presence of p62 in the Rab32 immunoprecipitate, suggesting a direct or indirect interaction between the two proteins (Fig. 1a) (quantification in Fig. S1, available in the online Supplementary Material).

To determine whether Rab32 and p62 co-localized on the SCV, we generated stable GFP-p62-expressing iBMDMs, infected them with *S*. Tm **Δ***gtgE***Δ***sopD2* and WT strains and carried out immunofluorescence analysis at the indicated time points (Fig. 1b). Our results demonstrated that GFP-p62 is recruited to the SCV at 1 h post-infection, with ~10% of SCVs being decorated with p62. The number of p62-positive vacuoles significantly decreased to 5% and 3% at 5 and 10 h post-infection, respectively (Fig. 1c). As shown previously, in cells infected with *S*. Tm **Δ***gtgE***Δ***sopD2,* Rab32 was found in 80% of the vacuoles (Fig. 1d) [21]. We did not observe any difference in GFP-p62 recruitment between cells infected with WT *S*. Tm or *S*. Tm **Δ***gtgE***Δ***sopD2,* suggesting that the presence of Rab32 on the SCV is not required for p62 recruitment. Only in 3% of the vacuoles did GFP-p62 and Rab32 co-localize (Fig. 1d).

While the role of Rab32 in *Salmonella* killing in macrophages is well known, whether p62 participates in macrophage host defence is less clear [4]. To address this, we knocked down p62 in macrophages (Fig. S2), and these cells were infected with *S*. Tm **Δ***gtgE***Δ***sopD2* or WT strains. Both *S*. Tm **Δ***gtgE***Δ***sopD2* and WT strains exhibited significantly higher survival in p62-deficient macrophages at 24 h post-infection compared with controls (Fig. 1e). This highlights the critical role of p62 in macrophage defence and suggests that, while both Rab32 and p62 play key roles in host defence, they may operate through distinct host-protective pathways.

### Recruitment of autophagy-related proteins to the SCV is independent of p62

**Fig. 2.**
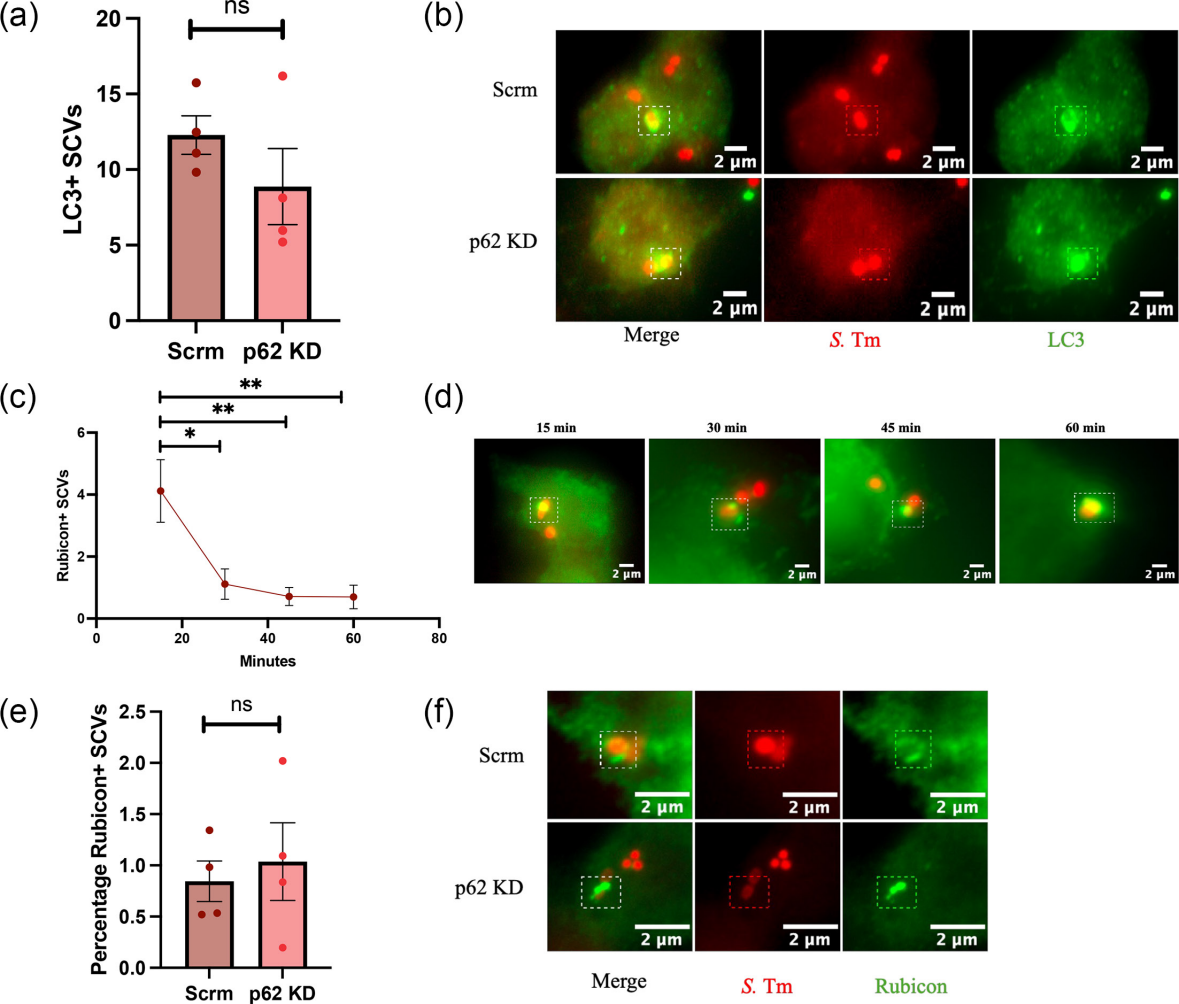
Depletion of p62 does not affect recruitment of autophagy proteins. (a) Quantification of vacuoles positive for LC3. iBMDM control (Scrm) and p62 KD cells were infected with mCherry *S.* Tm WT, fixed at 1.5 h post-infection, and stained with an anti-LC3 antibody. Data show mean±se of four independent experiments, paired t-test. (b) Representative images of LC3 staining. (c) Fixed WT iBMDMs infected with mCherry *S.* Tm were stained for Rubicon every 15 min post-infection. Data show mean±se of three independent experiments. *P<0.05; **P< 0.01, Mann–Whitney U test. (d) Representative images of Rubicon staining over 60 min. (e) Fixed WT iBMDMs infected with mCherry *S.* Tm were stained for Rubicon at 15 min post-infection. ns, not significant, Student’s t-test. Data show mean±se of four independent experiments. (f) Representative example of Rubicon-positive vacuoles.

Considering the role of p62 as an autophagy receptor, we hypothesized that the significant reduction in *S*. Tm killing within p62-depleted macrophages was due to impaired autophagy [6,22]. To test this, control and p62-depleted macrophages were infected with *S*. Tm WT, fixed at 1.5 h post-infection and stained for LC3 (Fig. 2a, b). No significant difference in bacteria positive for LC3 was observed (paired t-test, *P*=0.32) (Fig. 2a). These results indicate that the reduction in p62 has no impact on the recruitment of LC3 to the SCV. However, redundancy between intracellular autophagy receptors, like p62 and NDP52, may mask any observable differences in LC3 recruitment [23].

It is known that LC3-associated phagocytosis (LAP) is functional in macrophages, though whether p62 participates in this pathway remains unknown [24,25]. To address this, first, WT macrophages were infected with *S*. Tm WT and fixed every 15 min over a period of 60 min, and the recruitment of Rubicon (a protein found exclusively at the beginning of the LAP pathway) was assessed by immunofluorescence. Rubicon recruitment to the SCV was rare, found on only 4% of vacuoles at 15 min (Fig. 2e, f), and by 30 min post-infection, only 1% of *S*. Tm vacuoles were found to be positive, a significant reduction. Moreover, we did not observe a significant difference in Rubicon recruitment between WT and p62-depleted macrophages (Fig. 2c). These results suggest that p62 is not involved in the recruitment of Rubicon to the SCV in murine macrophages.

To summarize, our results indicate that the recruitment of canonical and non-canonical autophagy markers to the SCV in murine macrophages is independent of the presence of p62, suggesting that the role of p62 in controlling *Salmonella* infection goes beyond its role in autophagy.

### Depletion of p62 reduces the pro-inflammatory response of macrophages

p62 is known to interact with components of the TLR-4 signalling pathway, contributing to the induction of pro-inflammatory cytokines [7]. Based on this, we hypothesized that macrophages lacking p62 would exhibit a dampened pro-inflammatory response, thereby facilitating increased survival of *S*. Tm.

**Fig. 3.**
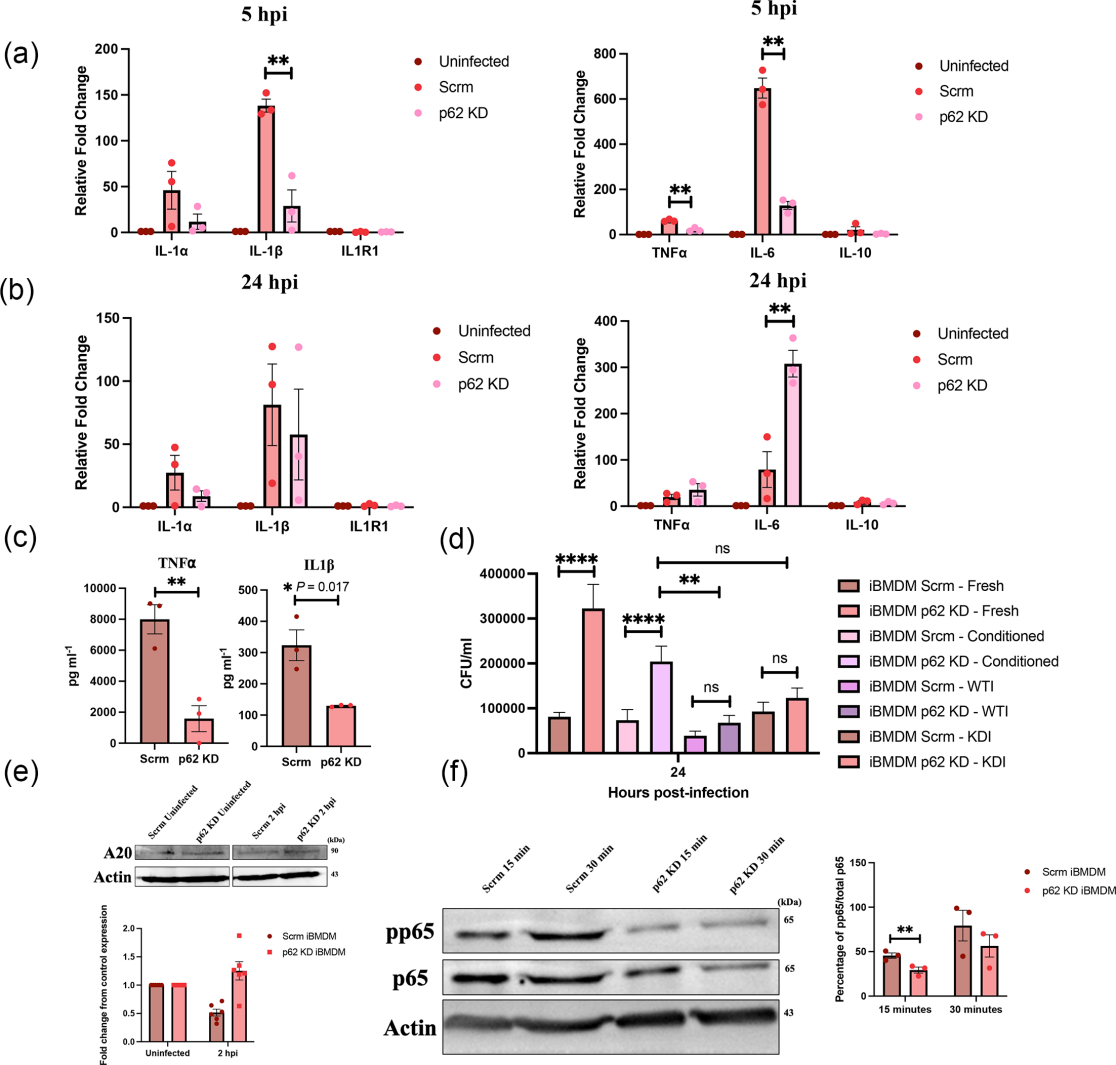
Depletion of p62 reduces the pro-inflammatory response of the macrophages. Relative fold change in IL-1⍺/β, IL1R1 (left), TNF⍺, IL-6 and IL-10 (right) gene expression, calculated by the 𝝙𝝙Ct method in control (Scrm) and p62 KD macrophages at 5 hpi (a) and 24 hpi (b) with *S.* Tm WT at an MOI of 5. A** = *P*<0.01, Student’s t-test. Data from three independent experiments, mean±sem. (c) ELISAs were performed to measure the protein concentrations of IL-1β (left) and TNF⍺ (right) in the supernatant of WT and p62 KD macrophages infected with WT *S.* Tm at an MOI of 5. *P<0.05, **P<0.01, Student’s t-test. Data show the average of three independent experiments, mean±sem. (d) Supernatant at 5 hpi was replaced with media from previously infected WT iBMDMs (WTI), p62 KD iBMDMs (KDI) or conditioned media. Lysates were plated onto LB agar at the marked time points and colonies counted. MOI = 5, ns = not significant, **P< 0.01, ***P<0.001, ****P<0.0001, Student’s t-test. Data show the average of three independent experiments, mean±sem. (e) Infected (MOI 5) and uninfected cell samples were lysed at 2 hpi and run through a 12% polyacrylamide gel to separate the proteins. (Left) The PVDF membrane was stained with an A20 antibody, representative of six independent experiments. (Right) Quantification of protein expression. *P< 0.05, ***P<0.001, Student’s t-test. Data show the average of six independent experiments, mean±sem. (f) Infected (MOI 5) cell samples were lysed at 15- and 30-min post-infection and run through a 12% polyacrylamide gel to separate the proteins. (Left) The PVDF membrane was stained with an anti-p65 and an anti-pp65, with actin as a loading control. Representative of three independent experiments. (Right) Quantification of protein expression, the percentage of pp65 in the total amount of p65. **P< 0.01, Student’s t-test. hpi, hours post-infection.

To test this, we assessed the expression of key cytokines (IL-1β, TNFα, IL-6 and IL-10) by RT-qPCR in control and p62-depleted macrophages infected with WT *S*. Tm. Cells were lysed at 5 and 24 h post-infection [26]. We observed a significant decrease in the expression of IL-1β, TNFɑ and IL-6 in p62-depleted macrophages compared with WT macrophages only at 5 h post-infection (Fig. 3a). In contrast, at 24 h post-infection, there was a significant increase in IL-6 levels in p62-depleted macrophages (Fig. 3b), highlighting differences in the signalling pathways downstream of these cytokine receptors.

To corroborate these findings at the protein level, ELISAs were performed to quantify IL-1β and TNFα in culture supernatants collected 24 h post-infection. (Fig. 3c). Consistent with transcript data, p62-depleted macrophages secreted significantly lower levels of these pro-inflammatory cytokines, which suggests that the increased survival of *Salmonella* in these cells could be a result of their inability to mount a pro-inflammatory response. To directly assess whether the reduced pro-inflammatory response contributed to increased bacterial survival, we performed a gentamicin protection assay. Supernatants from infected WT or p62-depleted macrophages were collected 24 h post-infection and added to newly infected macrophages, followed by quantification of intracellular *S*. Tm survival (Fig. 3d). Supernatants from infected WT macrophages significantly enhanced bacterial killing in p62-depleted cells, compared with conditioned media from uninfected cells (*P*=0.004). In contrast, supernatants from infected p62-depleted macrophages failed to promote bacterial clearance in p62 KD macrophages to a significant degree compared with the conditioned media (*P*=0.067). These findings support the hypothesis that p62 contributes to *Salmonella* restriction in macrophages by promoting a pro-inflammatory cytokine response necessary for effective bacterial clearance.

p62 promotes NFκB activation by recruiting and activating TNF receptor associated factor 6 (TRAF6) via its TB domain [13]. We hypothesized that the reduced expression of pro-inflammatory cytokines in p62-depleted macrophages was due to a decreased activation of NFκB. Western blot analysis showed a significant reduction in phosphorylated p65 (pp65; one of the subunits of NFκB) at 15 min post-infection (Fig. 3f). Additionally, we measured the levels of A20, a negative regulator of NFκB, by Western blot in both WT and p62-depleted macrophages infected with *Salmonella*. The results obtained showed that p62-depleted cells contained higher levels of the inhibitor, further suppressing NFκB signalling (Fig. 3e).

Taken together, these results suggest that depletion of p62 negatively affects the pro-inflammatory response of macrophages produced via NFκB, thereby reducing levels of activation and decreasing the production of pro-inflammatory mediators by the cell.

## Discussion

*Salmonella* Typhimurium remains a pathogen of global priority, and, therefore, understanding host immune mechanisms that restrict its intracellular survival is critical for developing new therapeutic approaches [1]. In this study, we demonstrate that in murine macrophages, p62 plays a crucial role in controlling *S*. Tm infection, acting upstream of the transcription factor NFκB.

p62 is a well-characterized autophagy receptor, linking polyubiquitinated cargo and LC3, facilitating autophagosome formation [27]. p62, NDP52 and Optineurin have all been previously reported to interact with *Salmonella* through K63-linked ubiquitin chains [23,28]. In non-professional phagocytic cells, *S*. Tm escapes the SCV into the cytoplasm and undergoes hyper-replication; under these conditions, interactions with p62 promote autophagy and bacterial clearance [10,27]. In contrast, within macrophages, where *S*. Tm typically remains enclosed in the SCV, both NDP52 and p62 have been shown to traffic independently to the SCV with similar kinetics to initiate autophagy [23]. Here, we have shown that the lack of p62 does not prevent recruitment of LC3 to the SCV nor of Rubicon, which suggests that p62 could drive bacterial killing in macrophages independent of autophagy.

Instead, our findings indicate that p62 is essential for mounting a robust pro-inflammatory response. In p62-deficient macrophages, we observed reduced expression and secretion of key cytokines, such as IL-1β, TNFα and IL-6, early during infection. This is consistent with the known role of p62 in facilitating TLR-4, IL-1R1 and TNF Receptor signalling pathways, all of which converge on the IKK complex to activate NFκB-dependent transcription [29]. The impaired cytokine production in p62-depleted macrophages likely disrupts both autocrine and paracrine signalling, ultimately allowing enhanced bacterial survival. Thus, p62 loss is affecting not only the initial production of these cytokines but also their autocrine and paracrine signalling effects.

Interestingly, we observed that p62-depleted macrophages had significantly more IL-6 gene expression compared with control cells at 24 h post-infection. This may be explained by the ability of IL-6 to signal through the JAK2–STAT3 axis, bypassing the TRAF6- and RIPK-1-dependent pathways to activate inflammatory gene expression independently of p62-mediated NFκB signalling [30,31].

TLR-4 has shown the capacity to signal intracellularly once endocytosed through the TRIF-TRAM pathway, signalling through RIPK-1, which, as previously described, is an interactor of p62 [32,33]. Therefore, it is possible that the small proportion of vacuoles that we observed positive for p62 (10%) is a result of p62 recruitment via RIPK-1 upon TLR-4 internalization. Additionally, it has been shown that TLR-4 traffics to the Golgi apparatus upon stimulation with LPS, where Rab32 is present [34]. At the Golgi, Rab32 is a necessary component of retromer complex-mediated recycling of plasma membrane components, including TLRs [35]. Thus, it is possible that the interaction observed between Rab32 and p62 occurs at the Golgi rather than on the SCV.

In summary, our study provides novel evidence showing that p62 is necessary to control *S*. Tm growth in murine macrophages, and we suggest that the mechanism by which it does so is not through its role as a Sequestosome-like Receptor, but rather through mediating NFκB signalling downstream of intracellular TLR-4.

## Supplementary material

10.1099/acmi.0.001102.v3 Uncited Supplementary Material 1.

## Abbreviations

BRAM: BLOC-3-Rab32 antimicrobial pathway
DMEM: Dulbecco’s Modified Eagle Medium
dNTP: deoxynucleotide triphosphates
HBSS: Hank’s Balanced Salt Solution
hpi: hours post-infection
HRP: horseradish peroxidase
iBMDMs: immortalized bone marrow-derived macrophages
IP: immunoprecipitate
KD: knockdown
LAP: LC3-associated phagocytosis
MOI: multiplicity of infection
PEI: polyethyleneimine
pp65: phosphorylated p65
SCV: *Salmonella*-containing vacuole
SPI: *Salmonella* pathogenicity island
*S.* Tm: *Salmonella enterica* serovar Typhimurium
TB: TRAF6-binding
TLR-4: Toll-like receptor 4
WCL: whole cell lysate
