# Supplementary material for: p62 limits Salmonella Typhimurium in macrophages through its role in cell signalling
Source: Access Microbiol. 2026 Jan 22;8(1):001102.v3. doi: 10.1099/acmi.0.001102.v3 (PMC12827565; doi:10.1099/acmi.0.001102.v3)
Supplement: Uncited Supplementary Material 1. [file acmi-8-01102-s001.pdf]

Supplementary

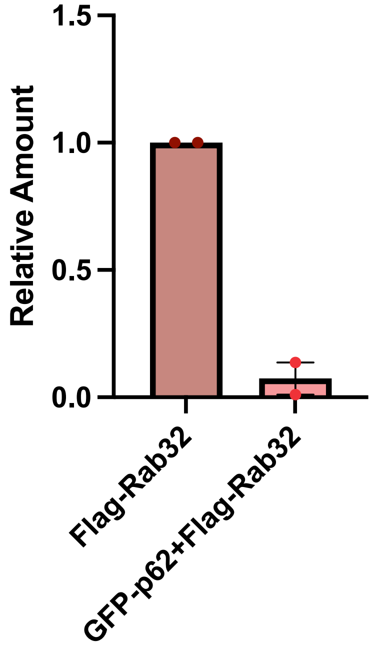

Supplementary Figure 1: Quantification of p62/Rab32 colocalization from immunoprecipitation

Quantification and relative fold change of the Flag-Rab32+GFP-p62 pull down, as represented in Fig. 1A. Data collected from two independent experiments

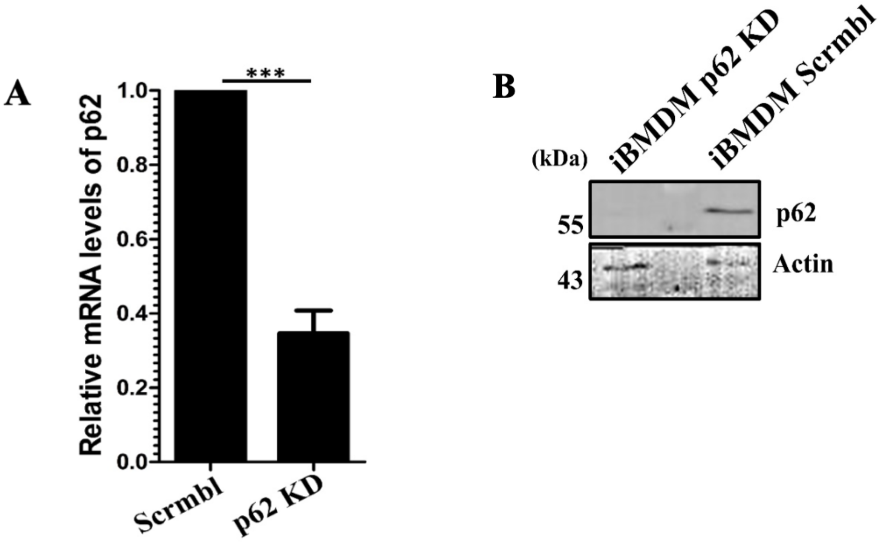

Supplementary Figure 2: Quantification of p62 levels in p62 KD iBMDMs

A) iBMDM cells were treated with a lentivirus encoding an shRNA targeting p62. Relative mRNA expression levels of 3 independent expression with standard deviation are shown. B) Western blotting was performed to confirm p62 knockdown at the protein level compared to control cells. *** = p<0.001, KD = Knockdown, Scrmbl = Scambled (control), kDA = kilodaltons
